# Consultant medical trainers, modernising medical careers (MMC) and the European time directive (EWTD): tensions and challenges in a changing medical education context

**DOI:** 10.1186/1472-6920-8-31

**Published:** 2008-05-20

**Authors:** Maria Tsouroufli, Heather Payne

**Affiliations:** 1 School of Medicine, Health Policy and Practice, University of East Anglia, Norwich, NR4 7TJ, UK; 2 Wales College of Medicine, School of Postgraduate Medical and Dental Education, Cardiff University, UK

## Abstract

**Background:**

We analysed the learning and professional development narratives of Hospital Consultants training junior staff ('Consultant Trainers') in order to identify impediments to successful postgraduate medical training in the UK, in the context of Modernising Medical Careers (MMC) and the European Working Time Directive (EWTD).

**Methods:**

Qualitative study. Learning and continuing professional development (CPD), were discussed in the context of Consultant Trainers' personal biographies, organisational culture and medical education practices. We conducted life story interviews with 20 Hospital Consultants in six NHS Trusts in Wales in 2005.

**Results:**

Consultant Trainers felt that new working patterns resulting from the EWTD and MMC have changed the nature of medical education. Loss of continuity of care, reduced clinical exposure of medical trainees and loss of the popular apprenticeship model were seen as detrimental for the quality of medical training and patient care. Consultant Trainers' perceptions of medical education were embedded in a traditional medical education culture, which expected long hours' availability, personal sacrifices and learning without formal educational support and supervision. Over-reliance on apprenticeship in combination with lack of organisational support for Consultant Trainers' new responsibilities, resulting from the introduction of MMC, and lack of interest in pursuing training in teaching, supervision and assessment represent potentially significant barriers to progress.

**Conclusion:**

This study identifies issues with significant implications for the implementation of MMC within the context of EWTD. Postgraduate Deaneries, NHS Trusts and the new body; NHS: Medical Education England should deal with the deficiencies of MMC and challenges of ETWD and aspire to excellence. Further research is needed to investigate the views and educational practices of Consultant Medical Trainers and medical trainees.

## Background

Structured training, clearly defined competencies, transparent assessment, and emphasis on self-directed and lifelong learning are key features of Modernising Medical Careers (MMC) [1]. This new scheme replaced the current medical training grades with a 2-year Foundation Programme followed by a 3–7 year specialty training programme [2]. It aimed to provide trainees with generic skills and experience in a variety of settings and specialities [3] leading to the award of a Certificate of Completed Training (CCT), described in the document 'The Next Steps' [4].

However, the recently published Tooke report (2008) recommended changes to MMC and in particular the return to a 1-year house officer training followed by a three-year broader-based training prior to higher specialist training. It also identified issues that hampered MMC. Lack of policy objectives; no guiding principles shared by all stakeholders; lack of clarity about the role of doctors; erosion of health-education partnership; lack of involvement of the medical profession in medical management, leadership and policy. The Tooke report also addressed the effect of the current interpretation of EWTD by UK legislation and the implications on the acquisition of clinical experience, confidence and the ability to shoulder responsibility [5].

Well before the publication of the Tooke report (2008) concerns regarding MMC had been expressed by both trainers and trainees [6], particularly whether the Foundation schemes will adequately prepare trainees for specialist programmes. There was also concern about the impact of MMC and EWTD on the quality of medical training, surgical training [7] in particular and the quality of patient care [8]. Fears had been expressed that the 'apprenticeship model' will be destroyed [9] and that shorter training will undermine the authority and status of the medical profession [10].

However, 'Next Steps' [4] stated that the apprenticeship model should not be abandoned but rather managed appropriately within the new training system and the requirements of the EWTD [11]. Unfortunately, when MMC was implemented Trainers and Trainees were not equipped with a clear plan for managing and maintaining the apprenticeship model in a context of reduced working hours. Consultant Trainers were also expected to undertake new responsibilities (assessment and educational supervision) under the new training system and to be supported in their new role [2]. Although, training in assessment, supervision and teaching was offered by Postgraduate Deaneries, most Consultant Trainers did not have a suitable job plan with an appropriate workload and time to develop trainees, nor were supported by an education team when MMC was implemented.

Recently, Postgraduate Medical Education and Training Board (PMETB), a further challenge for Consultant Trainers, have set high standards for Clinical and Educational Supervisors, giving attention to Trainers' competency and support in their role [12].

However, Consultant Trainers' views about postgraduate training, their new roles and their everyday experiences under the new training system and the constraints of EWTD have received less attention in research, and subsequently Continuing Professional Development (CPD) objectives.

In this paper we explore Consultant Trainers' views on postgraduate medical education and the implications of cultural changes, resulting from MMC and EWTD, aiming to identify impediments in the successful implementation of MMC, within a context of reduced working hours. In view of the potential confusion about the exact roles of the 'Educational' and 'Clinical' Supervisor we will use the generic term 'Consultant Trainer' throughout this paper.

## Methods

This qualitative, interview based study was conducted in 2005 on 20 practising NHS Hospital Consultants with responsibilities for supervising trainee Doctors, from six NHS Trusts in Wales. The study was funded by Cardiff University. This research study was conducted in compliance with the Helsinki Declaration [13]. It was reviewed and approved by MREC Wales (Ref. no. 05/MRE09/53). R&D approval was also granted by six NHS Trusts in Wales.

### Recruitment

Research participants were recruited from attendees at a short training course organised by the Postgraduate Deanery at Cardiff University (n = 13). Snowballing was also used. 7 research participants were recruited through interviewees' personal contacts and had not attended the training course. The course which looked at teaching, assessment and educational supervision under the new training system (MMC) was delivered at many sites across Wales and was attended by a total of 60 consultants. The Principal Investigator was a non-participant observer at the course delivered at Cardiff. Invitations to participate in the study were sent to all attendees. Those who returned their consent forms (20) were followed up by email or phone.

### Sample

The sample size was determined by saturation of data. This sample of consultants gave diversity of age (35 – 55), sex (11 female, 9 male), ethnicity (1 Asian, 2 Arab, 1 Greek, 3 English, 2 Irish, 1 Scot and 10 Welsh), and clinical specialty (general medicine, surgery, radiology, cardiology, obstetrics and gynaecology, ENT, paediatrics, emergency care, clinical pathology, anaesthetics, psychiatry). All research participants were committed educators who throughout their careers had been involved in formal or informal teaching, clinical and educational supervision and staff appraisal.

### Life-story approach and the interview schedule

A life story approach was employed because of its orientation towards exploring the importance of biography in interviewees' current views and practices [14]. The narrative approach gave interviewees the opportunity to discuss their own knowledge and experience of adult learning and professional development as complex processes, emerging from dynamic relationships between learners' personal biographies, organisational and professional culture and educational practices. Interviews contained discussion of MMC and the doctors' own medical teaching role. They also contained discussion of the interviewees' lifetime experiences of general and medical education. The themes discussed in interview were based upon literature in education [15] medical education [16,17] and the authors' clinical, educational and research experience. The interview schedule (table 1) was developed collaboratively and piloted with volunteers employed by the Postgraduate Deanery at Cardiff University.

**Table 1:** Interview schedule topics

Employing organisation
Interviewee's life and career
Experiences of learning
Experiences of teaching
Experiences of supervision
Continuing Professional Development

### Procedure

Interviews were conducted by the Principal Investigator – a female social scientist aged 35 – in Consultants' offices, a seminar room in the hospital or University. Consultants' unfamiliarity with qualitative research and their busy schedule were important challenges for the interviewer and the interviewees who seemed to hesitate to express opinions or feelings. Interviews lasted around an hour or more in some cases and were audio recorded, transcribed, anonymised and imported into the qualitative analysis package Nvivo 2.0. Field-notes were also written up after each interview, recording reflections on the interviews and initial analytical comments.

### Analysis

A record of analysis as well as detailed information about the research process and the participants is available in Nvivo 2.0. We adopted collaborative analysis and writing to maximise the confirmability and credibility of our findings [18]. The authors' different academic backgrounds (qualitative social scientist-clinician/medical educator) enriched the analytical process.

The interviews were coded by the PI under the broad descriptive categories (table 1) of the interview schedule with the purpose of easy retrieval of data in Nvivo 2.0. Authors worked independently and met regularly to discuss their thoughts on literal and interpretive reading of the data [18] Ideas emerged from these two analytical stages which were developed further during the last stage of the analysis; the reflective reading [19]. Authors were able to access, read and discuss each other's reflective notes in Nvivo 2.0. Through the process of discussion and comparison of data [20] and reading of relevant literature, the change of working practices resulting from EWTD and MMC; and Consultant Medical Teachers' traditional discourses of medical education emerged as key issues in our analysis.

The analysis presented in this paper was guided by three research questions:

• **What are Consultant Medical Teachers' perceptions of medical education?**

• **What are the links between organisational and educational changes?**

• **What is the relationship between personal experience of training (as a junior doctor) and current views?**

## Results

In what follows we explore Consultant Trainers' views about the cultural changes that have occurred as a result of MMC and EWTD. We also discuss Consultant Trainers' views of the effect of these changes on trainees' competency, Trainers' professional-educational role and Continuing Professional Development (CPD).

### Old 'apprenticeship' and the new EWTD

Either explicitly or implicitly, Consultant Trainers expressed positive attitudes towards the apprenticeship model of learning or some aspects of it such as

'*stability' *(Doctor 3).

They focused on familiarity with many clinical procedures and fast learning which occurred

*'simply by being there, asking questions and learning from mistakes' *(Doctor 11).

Sharing information and discussing decision-making approaches informally with senior staff throughout the disease trajectory was seen as conducive to learning. However, similar opportunities have disappeared since the introduction of EWTD and the disintegration of the firm.

The introduction of shift work has resulted in reduced availability of trainees, reduced contact between trainers and trainees and limited opportunities for communication and learning as the following excerpt illustrates:

*Now, if I feel someone could have improved the care they gave somebody and would like to talk about it, I'd have to try and make a formal appointment with them which makes it seem very big, and formal, or I'll say I'll just mention it in passing when I see them, and then I don't see them weeks could go by so time for that sort of pointed follow-up of an individual case care is very, very difficult. ... Continuity is very difficult, and if you are going to give a hot report on somebody's performance when you are not there when they are doing it, and then you don't see them until two weeks afterwards, you can't do that and I think that makes the learning more difficult. If it is not hot taking on the message, 'well what did you think about that, or that might have been another way to do' it doesn't mean very much if it is two weeks later*. (Doctor 12)

The limited availability of trainees has also resulted to reduced clinical exposure and experience. In the following excerpt a surgeon expresses concerns about the quality of surgical training, as reduced working hours have inadvertently resulted to reduced surgical experience.

*Hands on training, right ok I want to do the hands on training, I said I have 3 theatre sessions a week and so I want my FP2 to be with me in three theatre sessions so that I can give him the hands on training, that is my commitment in the bid. But, they can't be with me because they are on call for that day, they don't come to the theatre with me. Because they have been taking history examinations to all this emergency cases and then the next day they are off, they are not allowed to join me, even if they, er I mean voluntary, because they have been asked to go home. So, when my trainee is around I don't have the theatre. How am I going to give him the hands on training? *(Doctor 6)

New working patterns resulting from the EWTD have altered the nature of medical communities and had implications for the quality of medical training.

### Consultant Trainers' perceptions of MMC trainees

Consultant Trainers felt that cultural changes, resulting from EWTD and MMC, impinged on trainees' competency. All interviewees described current trainees as less confident and less able to work independently in comparison to themselves, however sound their theoretical knowledge, and thought that trainees required a lot more support and direction than themselves when they were being trained. Consultant Trainers felt that trainees were struggling with the application of theoretical knowledge in real life situations, due to their limited clinical exposure.

Consultant Trainers also felt that structured training (MMC) and reduced working hours is creating a generation of doctors unable to deal with the pressures and challenges of the medical profession as the following excerpt illustrates:

*Well they *(trainees) *go through a very structured process, you know the amount of time they spend, particularly when they are qualified, actually doing their job it's substantially theirs,, so when they have to, which they're not suppose to, but when they have to work, out of hours or work very intensively for a short period of time, I don't think they're really got that sort of experience that they can necessarily cope and some people, obviously do, but, I think a lot of them do find it quite stressful when they have to say look after three very sick patients all at the same time, you know, well I've heard in silence that they just can't cope. Whereas, in the old days it would be part and parcel of you training really and you would cope*. (Doctor 7)

The implications of shorter training, in particular the deficiencies of current trainees, dominated the interviews with Consultant Trainers, whereas reflection and evaluation of Consultant Trainers' teaching and supervision practices was a non-issue, irrespective of interviewees' age, gender, ethnicity and clinical speciality. The doctor in the following excerpt was the only interviewee who expressed concerns about her teaching practices and a need for adjustment to the new reality of reduced working hours and shorter medical training.

*So I think it's been pointed out that the change in working hours means a need for change in the way of teaching the trouble is from the experience I've had of teaching, is of that apprenticeship method, so I find it difficult to think about well how else do I go about getting this message across, when I can't do it the way that worked for me and it was a good method for me. I know it's not a good method for some people but it was a good method for me*. (Doctor 12)

### MMC-new roles and responsibilities for Consultant Trainers

Complaints about time constraints and increased workload, resulting from Consultant Trainers' involvement in MMC were common in the interviewees. The majority of interviewees perceived their new responsibilities, including educational supervision and assessment as conducive to trainees' learning and important for the effectiveness of MMC. However, all Interviewees expressed concerns about lack of time for performing teaching, supervision and assessment of trainees, due to clinical commitments and pressures from NHS employers. The doctor in the following excerpt expresses concerns about the implications of her supervision commitments arising from MMC and the lack of organisational support.

*Yeah, well the FP1's I've, I've put an application to get a FP1, um, I think it's, it's definitely the way forward but I don't think it's been particularly well thought through. I mean, I just supervise 1 FP2 at the moment and it takes an awful lot of time and to go through all the materials and, and to do it properly, I don't know how we're coping because if you're in speciality with maybe one or two you can but from next August I'll have 5 and it takes me about an hour a week to, if I'm doing it properly, to supervise that particular FP2 and go through all their materials and assessments with them and I don't know how it's going to be possible, we've got sort of, 15 of them to do. The level of support will drop because we just won't have the time to do it. We're not being given any extra consultant time to, to perform that role. (*Doctor 8)

### Consultant Trainers' educational expertise and perceived training needs

Consultants Trainers felt that changes in working patterns and medical training had implications for trainees as well as their employing organisation. However, further training in teaching, supervision and assessment was not identified as an appropriate response to the changes brought about by MMC and the EWTD. There was a general feeling that competency in these areas was acquired through experience and informal learning. Interestingly Consultants Trainers, who had formal training; a formal role in undergraduate of postgraduate Medical Education; and a strong role model as a teacher/mentor/supervisor; were more enthusiastic about addressing professional challenges resulting from MMC and EWTD.

The doctor in the following excerpt came from a family who valued good teaching and mentoring. He used to consult his mother, a teacher, on educational and professional development issues. After reflection and careful needs assessment, he decided to do a Bachelor of Education to address challenges, resulting from his involvement in the Foundation Program and specialist training.

*The FP2s have an appraisal every two months and certainly every 4 months. The GP trainee will need assessment every six months before they move on to their next placement, a specialist registrar will need an appraisal every twelve months before they move onto their next placement. So I had a need to produce various educational material and a lot of my personal education has been around improving my teaching technique so that I can deliver that better. So it hasn't been about learning old age psychiatry, it is learning how to teach about old age psychiatry in a different setting or a better setting or more effective*. (Doctor 1)

Consultant Trainers' accounts show limited perceived benefit of teaching and supervision for their educational and professional development and variation in perceptions of their supervisory role. All interviewees described themselves as facilitators of the trainees' learning but most thought that their main task as MMC educational supervisors was to organise learning events, discuss aims and objectives with the trainee and inform them of the learning opportunities in their department. Providing regular feedback on performance and progress through formal or informal learning events was felt to be desirable by fewer interviewees (and not always achievable, due to organisational constraints). Supporting trainees in identifying learning needs, providing pastoral care and career advice were the least frequently mentioned activities mentioned by interviewees.

### Consultant Trainers' Personal Experience of Training-The Sink or Swim Medical Education Culture

Consultant Trainers' preference for apprenticeship and resistance to training, pertinent to Consultants' new role, might be associated with their personal experience of medical training. Long hours' availability, personal sacrifices, emotional and physical robustness and strength, self-reliance and independence, were subtle expectations of medicine when Consultants were junior doctors. Consultant Trainers felt that current trainees should make similar sacrifices and accept that medicine is not a 9-5 job as the following excerpts illustrate:

*'to come out of their *(trainees) *way to learn... rather than thinking as a nine to five clerical assistant' *(doctor 13).

*You have to be organised, say for instance today I am not going to do anything I will keep this time to myself for studying or after five by boss is having an emergency gastrectomy and I will go and see that. If you go and see the elective emergency gastrectomy starting at six you have to sacrifice whatever activities you have in the evening*. (Doctor 6)

As junior doctors, Consultant Trainers had ample opportunity for informal learning due to different working patterns. However, in the old days, relationships were not always easy and some Consultant Trainers commented on difficulties in raising concerns and getting constructive criticism from their seniors as the following excerpt shows:

*I would have like to feel able to ask questions and if I wasn't sure or wanted something explained I thought I would get support but it was more about you don't do it like that, you should have done this and then there's like you know, you want to know why but that was never given, it was just everything you did was wrong*. (Doctor 8)

Consultant Trainers acknowledged the problems arising from unstructured postgraduate training with no protected time for learning, no **formal **educational supervision and lack of constructive criticism, impinging on their learning. However, all interviewees felt that their difficult experiences enabled them to develop into independent learners and hardworking consultants as the following excerpt illustrates:

*My 1st job was in the A&E department as a pre-registration house officer with four of us together who graduated together from medical school and not a great deal of supervision from anyone more senior, we could go to people if we did have problems but we generally tried to resolve the problems as far as we could amongst ourselves. Lots of experience, it probably moulded me for the future that every job I did involve hard work and quite a lot of independent problem solving and taking responsibility for your own education and learning*. (Doctor 4)

## Conclusion

Analysis of educational narratives of 20 Consultant Trainers indicates that the EWTD has led to working practices which have altered the Trainee-Consultant Trainer relationship. The introduction of shift duties was perceived by our interviewees, to have fragmented medical communities, and significantly reduced contact between trainers and trainees. Similar findings were reported in McKee's ethnographic study of senior house officers [21].

New working practices resulting from the EWTD have implications for medical training. Our qualitative data shows that limited clinical exposure of trainees, lack of continuity, and limited Consultant Trainer-Trainee contact at the workplace, has inadvertently led to the loss of 'apprenticeship' long used and still favoured by both trainers and trainees. This causes concern to the medical community and is seen as a threat to the profession. Further research is needed to explore shared understanding of 'apprenticeship', but there was common reference to 'see one, do one, teach one'.

The implementation of MMC within the context of EWTD has created new responsibilities and challenges for Consultant Trainers. Our study findings show that lack of organisational support for Consultant Trainers and management strategies that would balance clinical and administrative impinged on the quality of medical training and Consultants' educational role.

Lack of clarity about Consultants' educational role in combination with their resistance to further training might have also been a barrier to the successful implementation of MMC, within a context of reduced working hours. This qualitative study has identified Consultant Trainers' predominant assumptions about learning, embedded in a 'sink or swim' medical education culture [22]. Having received limited supervision and support as juniors and little training in teaching and supervision [23] as Consultants, interviewees seemed to be heavily reliant on the 'old apprenticeship', focusing mainly on the deficiencies of their trainees and the constraining environments in which they work and teach. In our study, interviewees did not often appear to use more advanced teaching skills such as seeking to diversify educational techniques in the face of insuperable obstructions (time and synchronicity constraints), engagement in shared and reciprocal learning practices [24], reflection and evaluation of their own teaching and supervisory practices [25]. This is a matter for concern in a sample explicitly committed to training. It is also a key issue that needs to be addressed as Consultant Trainers are now expected to be involved in creating a learning culture, and provide a level of supervision appropriate to the competence and experience of the trainee [12].

Establishing appropriate mechanisms for the selection, organisational and educational support of Trainers [12] and fostering strong links between health and theeducation sector are potentially ways forward [5]. Also promoting effective involvement of the medical profession in training policy-making could lead to clear, shared principles about the implementation and management of postgraduate training [5]. Further qualitative research is also needed to investigate the quality of postgraduate training as perceived and experienced by Trainers and Trainees.

## Abbreviations

NHS: National Health Service, MMC: Modernising Medical Careers, EWTD: European Working Time Directive, MREC: Multi-site Research Ethics Committee, R&D: Research and Development and CPD: Continuing Professional Development.

## Competing interests

Maria Tsouroufli has no competing interests.

Heather Payne is employed by a Postgraduate Medical Deanery.

## Authors' contributions

Maria Tsouroufli was principal investigator for the study. Heather Payne was co-applicant on the study research proposal and contributed to each stage of the study development, process, analysis and reporting.

## Pre-publication history

The pre-publication history for this paper can be accessed here:


